# Regional Relationship between Macular Retinal Thickness and Corresponding Central Visual Field Sensitivity in Glaucoma Patients

**DOI:** 10.1155/2017/3720157

**Published:** 2017-03-21

**Authors:** Chun-Hsiu Liu, Shirley H. L. Chang, Shiu-Chen Wu

**Affiliations:** Department of Ophthalmology, Chang Gung Memorial Hospital, Chang Gung University College of Medicine, No. 5, Fuhsing Street, Kweishan, Taoyuan 333, Taiwan

## Abstract

*Purpose*. To investigate the relationship between macular retinal thickness (MRT) and central visual field sensitivity (VFS) in patients with glaucoma. *Methods*. This retrospective study enrolled patients diagnosed with open-angle glaucoma. All study patients underwent Humphrey 10-2 visual field (VF) test and Spectralis spectral-domain optical coherence tomography (SD-OCT) exam for MRT measurement. *Results*. Sixty-eight eyes of 68 patients were examined. The correlation coefficients between VFS and MRT were 0.331 (*P* = 0.006) and 0.491 (*P* = 0.000) in the superior and inferior hemispheres, respectively. The average MRT in the eyes with abnormal 10-2 VF hemifields was significantly thinner than that in the eyes without abnormal hemifields in both hemispheres (*P* = 0.005 and 0.000 in the superior and inferior hemisphere, resp.). The average MRT values with an optimal sensitivity-specificity balance for discriminating the abnormal VF hemifield from the normal hemifield were 273.5 *μ*m and 255.5 *μ*m in the superior and inferior hemisphere, respectively. The area under the receiver operating characteristic curve was 0.701 in the superior hemisphere and 0.784 in the inferior hemisphere (both *P* < 0.05). *Conclusions*. MRT measured through SD-OCT was significantly correlated with central VFS. Lower MRT values might be a warning sign for central VF defects in glaucoma patients.

## 1. Introduction

Glaucoma is among the leading causes of blindness worldwide. It is a group of ocular diseases characterized by optic neuropathy associated with progressive thinning of the neuroretinal rim and loss of the retinal nerve fiber layer (RNFL) together with a particular pattern of visual field (VF) loss. Compared with standard automated perimetry, which is a functional and subjective test with greater intertest variability, optical coherence tomography (OCT) provides a highly qualitative, objective, and reproducible structural assessment of the optic nerve, RNFL, and macular thickness [1]. A previous study has reported that the correlation between peripapillary RNFL (pRNFL) thickness measured through OCT and visual function is high [2]. In addition, glaucomatous damage to the RNFL can precede future VF damage by up to 5 years [3]. OCT has been recognized as the most useful diagnostic tool in detecting early glaucoma among structural tests.

The macula contains more than 30% of the retinal ganglion cells and is vital for visual function [4]. There is growing evidence that early glaucoma can affect the macula and cause paracentral VF deficits [5, 6]. However, the routine VF test using the Humphrey field analyzer (HFA) with the Swedish interactive threshold algorithm (SITA) 24-2 or 30-2 programs has test points spaced 6 degrees apart, with only 4 test points placed within the central 8 degrees of the visual field. Paracentral scotomas in certain patients can be overlooked in the routine 24-2 or 30-2 VF tests because of relatively poor central VF sampling [7–9]. By contrast, the 10-2 VF program has 68 test points within the central 10 degrees and thus provides more detailed information in the central VF. However, performing both the 24-2 and 10-2 VF testing for every glaucoma case is time consuming. A more objective and efficient method for evaluating the macula damage associated with glaucoma is necessary.

Since glaucomatous damage of the macula can compromise central visual function, even in the early stage, measuring macular retinal thickness (MRT) through OCT in early glaucoma patients is imperative and provides several advantages: the macular area contains the highest densities of retinal ganglion cells; measurements of MRT exhibit less variability than those of the peripapillary RNFL do. However, the nature of macula damage in early glaucoma and its relationship with the central VF are poorly understood. Rolle et al. showed that there is a significant structure-function correlation between MRT and central VF sensitivity (VFS) [10]. However, they extracted 16 central VF test points from the 24-2 VF for central VFS, which provided only gross information on the central VF. Because of the clinical importance of the central VF, more detailed information about central visual function should be evaluated, and the correlation between structure and central VFS should be elucidated. Therefore, in this study, we compared the VFS of the divided zones in the Humphrey 10-2 VF with the corresponding zones of MRT by using posterior pole asymmetry analysis in Spectralis spectral-domain OCT (SD-OCT). The purpose of our study was to determine the localized structure-function relationship in each corresponding area of the macula. It is crucial to clarify the structure-function relationship in the central VF to obtain diagnostic information on central VF defect detection based on MRT in the divided zones.

## 2. Materials and Methods

### 2.1. Participants

This retrospective study was conducted between August 2014 and July 2015. Participants were enrolled from the Glaucoma Clinic of the Department of Ophthalmology, Chang Gung Memorial Hospital, Taoyuan, Taiwan. The study enrolled patients diagnosed with primary open-angle glaucoma or normal tension glaucoma. Patients with glaucomatous optic neuropathy on fundus examination and a mean deviation less than −20 dB on the 30-2 VF testing were included. The Institutional Review Board and Ethics Committee of Chang Gung Memorial Hospital approved this study, which adhered to the tenets of the Declaration of Helsinki.

All patients underwent comprehensive ophthalmic evaluation, including best-corrected visual acuity (BCVA) assessment, refraction, slit-lamp biomicroscopy, intraocular pressure measurement, central corneal thickness measurement, axial length (AL) measurement (AL-Scan Optical Biometer; Nidek, Japan), optic nerve head (ONH) evaluation and fundus examination, digital color fundus photography (Digital Non-Mydriatic Retinal Camera; Canon, Tokyo, Japan), VF testing using the Humphrey 30-2 SITA standard strategy (Carl Zeiss Meditec; Jena, Germany) and 10-2 SITA standard strategy, and retinal thickness measurement over the posterior pole through Spectralis SD-OCT examination (Heidelberg Retinal Engineering, Dossenheim, Germany). The worse eye from each patient was selected. All examinations were conducted within 6 months of SD-OCT examination. The exclusion criteria were BCVA lower than 20/40 in Snellen equivalents; a spherical equivalent refractive error higher than +6.00 or −10.00 diopters; an age younger than 20 or older than 80 years; previous intraocular surgery; ocular diseases other than cataract and glaucoma; diseases that could affect macular thickness, such as macular pucker, macular edema, drusen, and diabetic retinopathy; and unreliable 30-2 and 10-2 VF test results (fixation loss of >33%, false negative error or false positive error of >33%).

Glaucoma was diagnosed when the optic disc exhibited glaucomatous changes, such as localized or diffuse neuroretinal rim thinning of the ONH, a vertical cup-to-disc ratio asymmetry greater than 0.2, or RNFL defects corresponding to the glaucomatous VF defects. Glaucomatous VF defects were defined on the basis of the Humphrey 30-2 VF testing and confirmed through at least 2 reliable examinations for which 1 or more of the following criteria were met: a cluster of 3 or more nonedge points with a probability of less than 5%, including 1 point or more with a probability of less than 1%, on the pattern deviation map in at least 1 hemifield; a pattern standard deviation with a probability of less than 5%; and glaucoma hemifield test results outside the normal limits [11]. Central VF defects on the 30-2 VF was defined as the involvement of at least one of the central 12 cardinal points corresponding to the region in the central 10 degrees tested by the 10-2 VF test points with the threshold of which was depressed by an amount significant at *P* < 5%. The 10-2 VFs were classified as abnormal by applying the cluster rule: a cluster of 3 or more contiguous points (5%, 5%, and 1% or 5%, 2%, and 2%) within a hemifield on either total deviation or pattern deviation maps [8].

### 2.2. SD-OCT Measurements

The Spectralis SD-OCT device measured posterior pole retinal thickness within a 30° × 25° OCT volume scan centered on the fovea; this area was divided into an 8 × 8 mm grid, consisting of 3° × 3° squares. The average retinal thicknesses of the superior and inferior hemispheres for each grid, as well as the total retinal thickness, were calculated. Only high-quality scans with signal strengths of more than 15 dB were used for analysis.

### 2.3. Statistical Analysis

**Figure 1:**
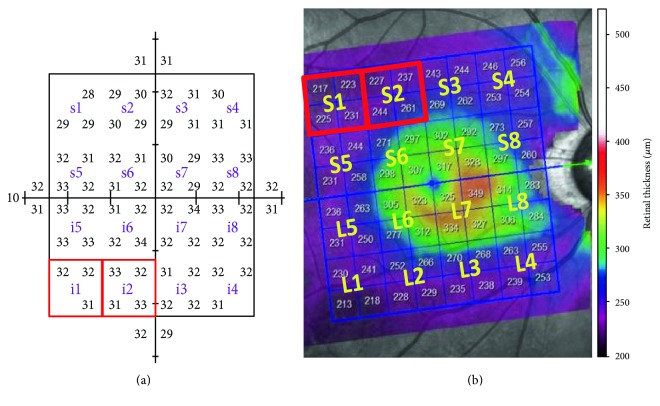
Divided corresponding zones in the macular retinal thickness map and Humphrey 10-2 test. (a) The 68 test points of the 10-2 test were divided into 8 zones in each hemisphere; the average threshold values in each zone were used for statistical analysis. (b) The posterior pole retinal thickness map was divided into 8 zones in each hemisphere. Average retinal thickness values of 4 adjacent square cells in each zone were used for statistical analysis. Each divided zone was labeled from 1 to 8 for each hemisphere. The average MRT of the superior temporal zone (S1) was matched with the VFS in the inferior nasal zone (i1), and the average MRT of the inferior nasal zone (i1) was matched with the VFS in the superior temporal zone (S1).

To analyze the structure-function relationship, we divided the posterior pole retinal thickness map and the 10-2 VF threshold map into 16 corresponding zones (Figure 1). The posterior pole retinal thickness map was divided into 8 zones in each hemisphere. Average retinal thickness values of the 4 adjacent square cells in each zone were used for statistical analysis. VF sensitivities were modified using the Lambert factor on the basis of the formula *dB* = 10∗log(1/L), and the average threshold values in each zone were used for statistical analysis. Each divided area of the posterior pole retinal thickness map was labeled from 1 to 8 for each hemisphere. The numbers were ordered from the temporal to the nasal retina and from the peripheral to the central retina. VFS values and SD-OCT data were all registered at the right eye orientation. The correlation between average MRT and VFS in each corresponding zone, as well as the average hemisphere and total average values, was evaluated. Superior MRT (S) was matched with inferior VFS (i); inferior MRT (L) was matched with superior VFS (s). Pearson correlation was used to express the relationship between MRT and VFS. We also divided all patients into 2 groups: eyes with abnormal 10-2 VFs within a hemifield and eyes without abnormal 10-2 VFs within a hemifield. Mann–Whitney test was used to compare MRT in the 2 groups. The areas under the receiver operating characteristic (AUROCs) curve were calculated to assess the power of MRT to discriminate the 10-2 VF involvement. The best cut-off values of MRT for predicting the 10-2 VF involvement with the optimal sensitivity-specificity balance were derived from the Youden index [12]. All statistical analyses were performed using SPSS software version 19.0 (SPSS, Inc., Chicago, Illinois, USA). Data were expressed as the mean ± standard deviation. A *P* value less than 0.05 was considered statistically significant.

## 3. Results

**Figure 2:**
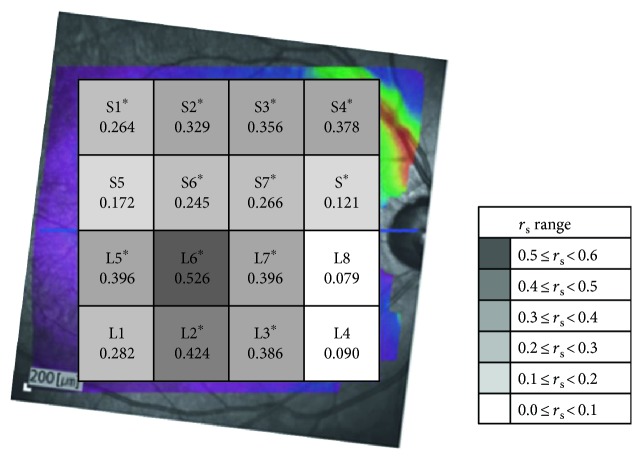
The correlation between visual field sensitivity and macular retinal thickness for 16 individual zones. The *r*_s_ ranges are shown in the bottom right in grayscale. ^∗^The correlation was significant (*P* < 0.05).

Sixty-eight eyes of 68 patients were examined in this study.  Table 1 summarizes the demographics and clinical characteristics. The mean deviation of the 30-2 VF was −6.93 dB.  Table 2 and Figure 2 show the structure-function correlations of the total MRT, hemisphere MRT, and MRT of the 16 divided zones, with the corresponding VFSs. The correlation coefficients between VFS and MRT were 0.331 and 0.491 in the superior and inferior hemispheres, respectively, and 0.079–0.526 in the divided zones. Significant correlations between MRT and VFS were found in both hemispheres and in most of the corresponding divided zones, particularly in the parafoveal areas (S1–S4, S6, S7, L2, L3, and L5–L7). The areas with higher correlation coefficients were located in the inferior parafoveal (L6 and L7), the inferior and temporal areas (L2, L3, and L5) in the inferior hemisphere, and the superior and nasal areas (S2–S4) in the superior hemisphere.

Table 3 demonstrates the agreement between the 30-2 VF and the 10-2 VF for central visual involvement. In particular, 10 (33.3%) of the 30 eyes for the inferior hemifield and 10 (20.0%) of the 50 eyes for the superior hemifield were classified as abnormal on the 10-2 VF but normal on the 30-2 VF. The MRT values of the eyes classified as normal on the 30-2 VF but abnormal on the 10-2 VF were significantly thinner than those of the eyes classified as normal on both the 30-2 VF and the 10-2 VF (267.20 ± 10.72 *μ*m versus 278.19 ± 16.76 *μ*m, *P* = 0.044 for the superior hemisphere and 265.10 ± 14.76 *μ*m versus 277.55 ± 12.76 *μ*m, *P* = 0.013 for the inferior hemisphere).

**Figure 3:**
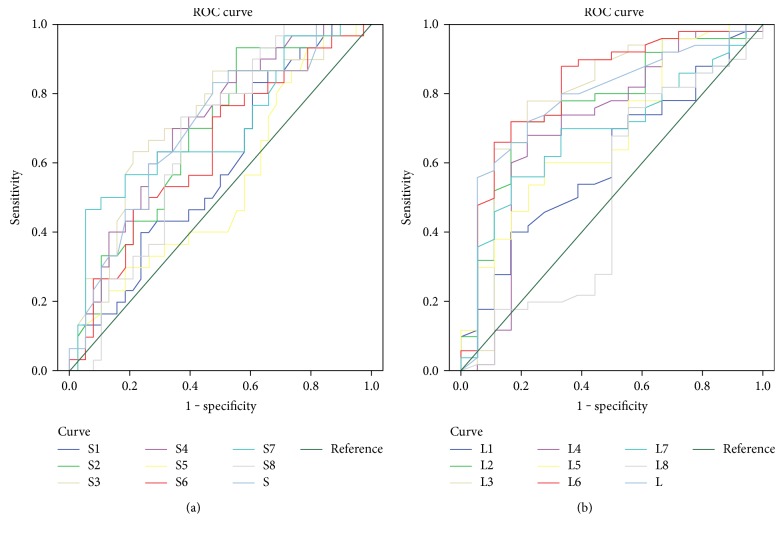
AUROCs of macular retinal thickness values for each divided zone. (a) ROC curve of MRT in the superior divided zones (S1–S8) and average MRT in the superior hemisphere (S). (b) ROC curve of MRT in the inferior divided areas (L1–L8) and average MRT in the inferior hemisphere (L).

Table 4 illustrates the differences in average MRT in the divided zones and hemispheres between the eyes with and those without abnormal 10-2 VF hemifields. The average MRT in the eyes with abnormal 10-2 VF hemifields was significantly lesser than that of the eyes without abnormal hemifields in both hemispheres and in most of the divided zones, except S1, S5, S6, L1, and L8, which comprised the more temporal peripheral areas. The AUROC and best cut-off values derived from the Youden index with optimal sensitivity-specificity balances for MRT values are listed in Table 5. To discriminate normal and abnormal VFs, the sensitivity and specificity with a cut-off value of 273.5 *μ*m in the superior hemisphere were 83.3% and 52.6%, respectively; the sensitivity and specificity with a cut-off value of 255.5 *μ*m in the inferior hemisphere were 56.0% and 94.4%, respectively. The discriminating power for central VF involvement was generally fair (AUROC range: 0.607–0.819) except in zone S1, S5, and L8. The diagnostic power of MRT was best in the inferior temporal parafoveal area (AUROC = 0.819, *P* = 0.000 in zone L6). The ROC curves in each zone were joined in a single chart as shown in Figure 3.

## 4. Discussion

In the present study, we demonstrated the structure-function relationship between MRT and central VFS. To the best of our knowledge, this is the first study to evaluate the regional correlation between MRT and central VFS by using the Spectralis SD-OCT device and Humphrey 10-2 VF test. The MRT values were shown to be significantly correlated with central VFS. Lower MRT values might be a warning sign for central VF deficits in early to moderate glaucoma.

The essential pathologic process in glaucoma is the loss of retinal ganglion cells and their axons, leading to a reduction in the thickness of the nerve fiber layer [13]. The loss of retinal ganglion cells and the nerve fiber layer can occur in the posterior pole, where these cells comprise 30%–35% of the retinal thickness. Macular thickness is a surrogate indicator of retinal ganglion cell thickness for glaucoma diagnosis [14]. Zeimer et al. first described losses in retinal thickness at the posterior pole of patients with early glaucoma by using a retinal topographer (Retinal Thickness Analyzer; Talia Technology Ltd., Neve Ilan, Israel) [15]. After the introduction of time-domain OCT, Greenfield et al. also reported reduced macular thickness in early- and moderate-stage glaucoma, and the changes in macular thickness were shown to correlate highly with visual function [14]. The newer generation of OCT, namely SD-OCT, enables an increased speed of image acquisition and improvements in eye tracking and signal-to-noise ratio, providing higher-resolution images and revealing larger areas of the macular region. By using SD-OCT with 3D OCT, Nakatani et al. demonstrated that macular thickness was significantly correlated with VFS in early glaucoma [16]. In addition, they found that the macular parameters of SD-OCT had higher reproducibility than those of pRNFL. Because pRNFL measurement is prone to be affected by misalignments and disc anomalies such as tilted discs and peripapillary atrophy, and because fixation is relatively easier for macular scans, macular thickness measurements may be more reproducible than pRNFL parameter measurements for glaucoma diagnosis. Ohkubo et al., by using SD-OCT with 3D OCT, further considered the thickness of the RNFL, ganglion cell layer (GCL), GCL and inner plexiform layer (IPL), and RNFL, GCL, and IPL (GCC) in the macular area. They found that the correlation with VFS was best to use in the GCL or GCL + IPL in the central 5.8 degree [17]. Ohkubo et al. claimed that the GCC is the most sensitive predictor for detecting macula damage.

In agreement with the aforementioned previous studies, we observed positive correlations between MRT and central VFS in most of the divided zones, particularly in the parafoveal area. The zones with lower correlations were located more peripherally or closer to the optic disc. The thickness of the areas close to the disc may have interfered with the peripapillary atrophy variables. The use of the 10-2 VF for central VF testing is unique in our study because it more accurately represents visual function in the macula. There is growing evidence that early glaucomatous damage involves the macula and causes corresponding central field change [5, 6]. However, because most previous studies used the 24-2 VF for central field testing and extracted only 16 central VF test points for structure-function correlation, the central field defects may have been underdetected as a result of poor spatial sampling [18]. By using the 10-2 VF, this study provided integrated VFS in the central VF, thus achieving higher accuracy in defining the structure-function relationship. This study shows significant structure-function correlations in most of the divided zones, particularly in the parafoveal area, with comparative correlation coefficients to previous study using the 24-2 VF [10]. Furthermore, this study not only demonstrates the central VF deficits might be overlooked by routine 30-2 VF in as high as 33% of eyes but also reveals the important role of MRT in detecting central VF deficits which might be missed by 30-2 VF.

The areas with significant different MRT between normal and abnormal VFS were L2–L4 and L5–L7 in the inferior hemisphere and S2–S4, S7, and S8 in the superior hemisphere. Hood et al., by using frequency-domain OCT, proposed a schematic model, termed the macular vulnerability zone, to describe the glaucomatous damage of the macula [5, 6, 9]. In their model, the inferior macula was more susceptible to glaucomatous arcuate damage, and the macular vulnerability zone was narrower close to the disc and wider close to the temporal parafoveal zone. Furthermore, the vulnerable zone in the superior hemisphere was farther from the macula compared with that in the lower hemisphere. This model facilitates explaining why in the present study, greater differences in macular thickness between normal and abnormal VF hemispheres were found in the inferior and temporal parafoveal area and outer zones of the superior hemisphere.

The AUROC for MRT was greater in the inferior hemisphere than that in the superior hemisphere, particularly in the inferior temporal parafoveal area; this is in agreement with previous studies. By using the device employed in the present study (i.e., the Spectralis OCT), Rolle et al. divided the posterior pole into 4 quadrants and identified the highest discriminating power in the inferior nasal quadrant (AUROC = 0.82) [10]. Dave et al. also found the highest AUROC for the average inferior macular thickness (AUROC = 0.833) [19]. However, in these 2 studies, the 24-2 VF was used for central field sensitivity. Nakatani et al., by using SD-OCT with 3D OCT, found significant differences for all macular parameters in glaucoma patients and healthy participants, with the highest AUROC for the temporal outer macular thickness (AUROC = 0.79) [16].

There were several limitations to our study. First, the exact correspondence of central VF points and macular thickness measurement points remained uncertain and the OCT measurement area is slightly wider than that of the 10-2 VF. Although the VF and MRT maps had similar visual angles, the reciprocal matching points of the 2 measurements may have differed in the absolute position [20]. Moreover, considering the morphologic displacement of retinal ganglion cells in the fovea area, correction for retinal ganglion cell displacement may be required to improve the focal structure-function correlation [17, 21]. However, there is currently no commercial OCT device that accounts for the displacement of retinal ganglion cells. In addition, taking the retinal ganglion cell displacement into account, the real anatomical areas in OCT corresponding to stimulation location of VF point would be wider than the extent of VF degree. It is thus reasonable to match the area of the 10-2 VF with posterior pole macular thickness in Spectralis OCT as shown in the present study. Second, only ethnically Chinese patients from Taiwan were analyzed in this study; differences may exist among ethnic groups. Another limitation is that a healthy group was not enrolled for evaluating the diagnostic power of MRT. Instead, we compared the MRT of patients with abnormal hemifields with that of patients without abnormal hemifields. Therefore, subtle structural changes may have remained to some extent in patients without abnormal hemifields, and the AUROC values in our study are slightly lower than those in a previous study [10]. Besides, the reliability criteria for VF testing were less strict than the reliability parameters of Humphrey Instruments Inc. However, some authors have recommended that relaxing the fixation loss criterion to less than 33% cut-off might increase the percentage of fields graded reliable with minimal effect on the sensitivity or specificity of the test [22].

The Spectralis SD-OCT device provides certain advantages: TruTrack active eye tracking and Heidelberg noise reduction enable acquiring accurate, reproducible, and high-quality maps, and the posterior pole analysis covers an area as wide as 8 × 8 mm in the macula. Furthermore, this device measures the total retinal thickness at the macular area, including layers which are not affected by glaucoma, rather than measuring the retinal ganglion cell complex, as in other OCT devices. Therefore, the Spectralis SD-OCT device may be relatively less sensitive to glaucomatous change but is less prone to segmentation error and allows for higher reproducibility.

In conclusion, our study revealed significant correlations between regional MRT and central VFS. The reduction of retinal thickness at the macular area was associated with the loss of the central VF in early- and moderate-stage glaucoma patients. Lower MRT values might be a warning sign for central VF defects which is easily missed when clinicians perform only standard perimetry with the 24-2 or 30-2 VF.

## Figures and Tables

**Table 1:** Demographic and ocular characteristics of the study participants.

	Total (*n* = 68)
Age (year)	54.73 ± 12.39
Male/female (number)	48/20
Intraocular pressure (mmHg)	13.67 ± 2.25
Spherical equivalents (diopters)	−4.07 ± 4.12
Axial length (mm)	25.52 ± 1.72
*Standard automatic perimetry*	
MD (dB)	−6.93 ± 4.64
PSD (dB)	8.22 ± 4.64

Data are presented as mean ± standard deviation.

MD: mean deviation; PSD: pattern standard deviation.

**Table 2:** Pearson correlation (*r*) between VFS and MRT for total values, matching hemispheres, and 16 divided zones: the superior MRT (S) was matched with the inferior VFS (i); the inferior MRT (L) was matched with the superior VFS (s).

Sectors	*r*	*P*	Sectors	*r*	*P*
S1/i1	0.264	0.030^∗^	L1/s1	0.282	0.202
S2/i2	0.329	0.006^∗^	L2/s2	0.424	0.000^∗^
S3/i3	0.356	0.003^∗^	L3/s3	0.386	0.001^∗^
S4/i4	0.378	0.001^∗^	L4/s4	0.090	0.464
S5/i5	0.172	0.160	L5/s5	0.396	0.001^∗^
S6/i6	0.245	0.044^∗^	L6/s6	0.526	0.000^∗^
S7/i7	0.266	0.028^∗^	L7/s7	0.396	0.001^∗^
S8/i8	0.121	0.325	L8/s8	0.079	0.522
S/i	0.331	0.006^∗^	L/s	0.491	0.000^∗^
Total	0.374	0.002^∗^			

^∗^ *P* < 0.05, MRT: macular retinal thickness; VFS: visual field sensitivity.

**Table 3:** Number of abnormal and normal hemifields on the 30-2 and 10-2 visual fields and hemisphere macular retinal thickness.

30-2	10-2				*P* value
	Normal (number)	MRT (*μ*m)	Abnormal (number)	MRT (*μ*m)	
*Upper visual field*					
Normal	11	277.55 ± 12.76	10	265.10 ± 14.76	0.013^∗^
Abnormal	7	260.14 ± 13.08	40	253.55 ± 13.85	0.178
Total	18	270.78 ± 15.25	50	255.86 ± 14.64	0.000^∗^
*Lower visual field*					
Normal	27	278.19 ± 16.76	10	267.20 ± 10.72	0.044^∗^
Abnormal	11	274.27 ± 21.85	20	264.25 ± 13.53	0.244
Total	38	277.05 ± 18.16	30	265.23 ± 12.55	0.005^∗^

Data are presented as mean ± standard deviation.

MRT: macular retinal thickness, ^∗^*P* < 0.05.

**Table 4:** Comparison of MRT between the eyes with and without an abnormal 10-2 VF hemifield: superior MRT (S); inferior MRT (L).

MRT (*μ*m)	Abnormal inferior VF hemifield (*n* = 30)	Normal inferior VF hemifield (*n* = 38)	*P*	MRT (*μ*m)	Abnormal superior VF hemifield (*n* = 50)	Normal superior VF hemifield (*n* = 18)	*P*
S1	227.07 ± 9.39	231.53 ± 12.02	0.224	L1	218.46 ± 11.28	223.01 ± 10.76	0.180
S2	248.91 ± 11.92	258.12 ± 15.92	0.009^∗^	L2	235.11 ± 14.00	248.90 ± 14.13	0.001^∗^
S3	260.59 ± 18.61	273.13 ± 19.44	0.002^∗^	L3	243.96 ± 17.17	264.01 ± 24.55	0.000^∗^
S4	259.87 ± 23.33	280.29 ± 27.24	0.003^∗^	L4	238.34 ± 39.34	243.83 ± 82.67	0.011^∗^
S5	248.05 ± 12.05	251.36 ± 15.35	0.630	L5	242.13 ± 11.59	250.44 ± 12.01	0.023^∗^
S6	294.30 ± 15.58	303.82 ± 23.24	0.052	L6	284.05 ± 13.90	302.36 ± 15.95	0.000^∗^
S7	305.07 ± 17.72	320.63 ± 26.98	0.006^∗^	L7	302.99 ± 20.72	314.47 ± 18.34	0.022^∗^
S8	247.67 ± 45.07	262.96 ± 78.60	0.020^∗^	L8	252.12 ± 62.62	240.68 ± 84.05	0.989
S	265.23 ± 12.55	277.05 ± 18.16	0.005^∗^	L	255.86 ± 14.64	270.78 ± 15.25	0.000^∗^

Data are presented as mean ± standard deviation.

MRT: macular retinal thickness; VF: visual field, ^∗^*P* < 0.05.

**Table 5:** AUROC value, 95% confidence interval, significance, optimal sensitivity-specificity balance, positive likelihood ratio (+LR), and negative likelihood ratio (−LR) for total MRT values and values for matching hemispheres, the 16 divided zones (MRT in superior hemisphere (S); MRT in superior hemisphere (L)), and matching quadrants.

Sectors	AUROC	95%CI	*P*	Best cut-off value (*μ*m)	Sensitivity	Specificity	+LR	−LR
Total	0.635	0.472 ~ 0.798	0.159	268.0	0.649	0.727	2.38	0.48
S	0.701	0.576 ~ 0.826	0.005^∗^	273.5	0.833	0.526	1.76	0.32
S1	0.586	0.451 ~ 0.722	0.224	232.6	0.833	0.395	1.38	0.42
S2	0.686	0.560 ~ 0.811	0.009^∗^	261.1	0.933	0.447	1.69	0.15
S3	0.722	0.597 ~ 0.847	0.002^∗^	259.6	0.633	0.789	3.01	0.46
S4	0.710	0.588 ~ 0.832	0.003^∗^	267.4	0.700	0.658	2.05	0.46
S5	0.534	0.394 ~ 0.674	0.630	263.0	0.933	0.211	1.18	0.32
S6	0.638	0.505 ~ 0.771	0.052	301.8	0.767	0.500	1.53	0.47
S7	0.697	0.567 ~ 0.827	0.006^∗^	297.3	0.467	0.947	8.87	0.56
S8	0.665	0.536 ~ 0.794	0.020^∗^	277.6	0.733	0.632	1.99	0.42
L	0.784	0.659 ~ 0.909	0.000^∗^	255.5	0.560	0.944	10.08	0.47
L1	0.607	0.461 ~ 0.754	0.180	216.4	0.400	0.833	2.4	0.72
L2	0.764	0.635 ~ 0.893	0.001^∗^	241.1	0.720	0.778	3.24	0.36
L3	0.784	0.641 ~ 0.928	0.000^∗^	256.6	0.780	0.778	3.51	0.28
L4	0.702	0.543 ~ 0.861	0.011^∗^	249.6	0.680	0.778	3.06	0.41
L5	0.682	0.540 ~ 0.824	0.023^∗^	245.8	0.600	0.722	2.16	0.55
L6	0.819	0.698 ~ 0.940	0.000^∗^	290.9	0.720	0.833	4.32	0.34
L7	0.683	0.547 ~ 0.819	0.022^∗^	303.8	0.560	0.833	3.36	0.53
L8	0.499	0.326 ~ 0.672	0.989	282.5	0.760	0.444	1.37	0.54

^∗^ *P* < 0.05, MRT: macular retinal thickness.
